# *TLR1* polymorphism rs4833095 as a risk factor for IgA nephropathy in a Chinese Han population: A case-control study

**DOI:** 10.18632/oncotarget.12965

**Published:** 2016-10-28

**Authors:** Jie Gao, Linting Wei, Jiali Wei, Ganglian Yao, Li Wang, Meng Wang, Xinghan Liu, Cong Dai, Tianbo Jin, Zhijun Dai, Rongguo Fu

**Affiliations:** ^1^ Department of Nephrology, Second Affiliated Hospital of Xi'an Jiaotong University, Xi'an 710004, China; ^2^ Department of Nephrology, Hainan general hospital, Haikou 570311, China; ^3^ Department of Oncology, Second Affiliated Hospital of Xi'an Jiaotong University, Xi'an 710004, China; ^4^ National Engineering Research Center for Miniaturized Detection Systems, School of Life Sciences, Northwest University, Xi'an 710069, China

**Keywords:** toll-like receptor 1, IgAN, single nucleotide polymorphism, susceptibility, case-control study

## Abstract

Toll-like receptors (TLRs) are a family of transmembrane receptors, and play a vital role in recognizing invading pathogens and activating innate immunity. Previous studies indicated that *TLR1* single nucleotide polymorphisms (SNPs) might be associated with the risk of IgA nephropathy (IgAN). This study aims to investigate the relationship between *TLR1* SNPs (rs4833095 and rs5743557) and IgAN in a Chinese Han population. This case-control study included 351 patients with IgAN and 310 healthy controls. Two SNPs (rs4833095 and rs5743557) of *TLR1* were genotyped by Sequenom MassARRAY. Odds ratios (OR) with 95% confidence intervals (CI) were used to assess the relationship with IgAN. We found that both allele and genotype frequencies of rs5743557 were not associated with IgAN risk. Rs4833095 increased IgAN risk compared with controls in the allele, dominant and log-additive models (*P* = 0.04, 0.04 and 0.03, respectively). Further haplotype analysis revealed that the T_rs4833095_T_rs5743557_ haplotype may be a risk factor for IgAN (OR = 1.28; 95% CI = 1.01–1.63; *P* = 0.046). Furthermore, rs4833095 was associated with Lee's grades (OR = 1.75; 95% CI = 1.03–2.96; *P* = 0.04). However, there was no significant association between the genotype distributions of rs5743557 and clinical parameters of IgAN such as gender, 24 hour urine protein, blood pressure, and Lee's grades. Taken together, these findings suggest that the *TLR1* rs4833095 polymorphism may play a role in the development and progression of IgAN.

## INTRODUCTION

IgA nephropathy (IgAN) is a common form of primary glomerulonephritis worldwide [1] and was first described in 1968 by Berger [2]. The diagnosis of IgAN depends entirely on renal biopsies. Painless gross hematuria is very common in children and adolescents and often coincides with, or immediately follows mucosal infections, especially of the upper respiratory tract. It is widely acknowledged that, in patients with IgAN, the mesangium IgA deposits are exclusively of the IgA1 subclass and often present with abnormal glycosylation [3, 4]. This phenomenon has been found to be the consequence of abnormal systemic responses to mucosally encountered antigens in IgAN patients [5]. Mucosal immunity is often triggered by receptors that can recognize pathogen associated molecular patterns expressed in pathogens of bacterial, viral, and fungal origins [6]. Among this, Toll-like receptors (TLRs) are the most frequent pattern recognition receptors [7–9].

TLRs are transmembrane receptors that play a vital role in recognizing invading pathogens [10] and activating innate immunity. Ten functional TLRs have been described in humans to date [11]. *TLR1* cooperates with *TLR2* to form the *TLR1*/*TLR2* heterodimer, which participates in a signaling cascade by stimulating innate and adaptive immune reactions in response to microbial agonists [12]. This heterodimer can recognize a variety of lipoproteins, including those from mycobacteria and meningococci [13].

Previous studies most focused on the relationship between *TLR4, TLR 9* and *TLR 10* with IgAN. However, some also investigated gene variants of *TLR1* with other diseases. For example, a study in Korea by Seok et al. reported that *TLR1* rs4833095 may be correlated with alopecia areata, and rs5743557 was also weakly linked with the development of alopecia areata [14]. Yang et al. found that rs4833095 polymorphism was predisposition to allergic diseases in Taiwanese population [15]. Besides, Thompson et al. suggested that rs4833095 variant was associated with increased mortality in sepsis patients after traumatic injuries [16]. Tang et al. [17] Ravishankar et al. [18] and Tongtawee et al. [19] demonstrated that *TLR1* rs4833095 polymorphism related to H. pylori and predisposition to gastric lesions. *TLR1* rs4833095 was also linked with risk of Crohn's and ulcerative colitis diseases [20]. Functional studies by Dittrich et al. showed the association of rs4833095 with tuberculosis protection in India [21]. But recent meta analysis by Schurz et al. indicated that rs4833095 variant was not correlated with tuberculosis [22]. In light of the controversial results, the precise association between *TLR1* gene polymorphisms with IgAN need studied.

Only one of these studies assessed *TLR1* gene polymorphisms with IgAN risk. And the study indicated that *TLR1* polymorphisms may be risk factors for IgAN in Korean children [23]. However, the precise pathogenesis has not been elucidated. We conducted this case-control study to determine the association between *TLR1* polymorphisms (rs4833095 and rs5743557) and IgAN and assess whether *TLR1* contributes to the risk and/or development of IgAN in the Chinese Han population.

## RESULTS

### Demographic and clinical features of the study population

This study involved 661 subjects, including 351 patients (229 males and 122 females; age at diagnosis: 32 ± 11.9 years) and 310 healthy controls (186 males and 124 females; age: 35 ± 12.6 years). The genotype frequency distributions of two selected SNPs (rs4833095 and rs5743557) in control subjects were in Hardy-Weinberg equilibrium (*P* = 0.94, and *P* = 0.80, respectively). The basic features of the subjects such as gender, age, and blood pressure are listed in Table 1. Hypertension was defined as blood pressure ≥ 140/90 mmHg on three occasions at diagnosis, or the use of antihypertensive medication to achieve normal blood pressure. 24 hour urine protein was divided into two groups, < 3.5 and ≥ 3.5 g. Lee's grade was used in two groups as follows: I + II + III and IV + V. There was no significant difference in age or gender between the case and control groups (*P* = 0.16 and *P* = 0.45, respectively, Table 1).

**Table 1:** Demographical and clinical informations of study subjects

Characteristics		Case	Control	*P* value
**Number**		351	310	
**Age (mean ± SD)**		32±11.9	35±12.6	0.45^a^
**Gender**				
**Male**		229	186	
**Female**		122	124	0.16^b^
**SCr (μmol/L)**				
**(mean ± SD)**		159.5±146.0		
**BUN (mmol/L)**				
**(mean ± SD)**		8.2±5.9		
**ALB (g/L)**				
**(mean ± SD)**		34.0±7.9		
**Serum IgA (g/L)**				
**(mean ± SD)**		2.8±1.7		
**Serum C3 (g/L)**				
**(mean ± SD)**		1.1±0.3		
**Cho (mmol/L)**				
**(mean ± SD)**		6.1±1.4		
**Urine protein**	<3.5	272		
**(g/24h)**	≥3.5	79		
**Blood Pressure (mmHg)**	<140/90	194		
	≥140/90	157		
**Lee's grades**	I+II+III	260		
	IV+V	91		

a P values was calculated from two-sided chi-square test;

b P values were calculated by Student *t* tests.

SCr = Serum Creatinine, BUN = Blood Urea Nitrogen, ALB= Serum Albumin, Cho = Cholesterol.

### The associations between *TLR1* SNPs and IgAN

The allele and genotype frequency distributions of the two *TLR1* SNPs in patients with IgAN and healthy controls are listed in Table 2. T allele frequencies of rs4833095 and rs5743557 in IgAN groups were 40.2% and 48.9%, respectively. Chi-square tests were performed under the assumption that the minor allele of each SNP was the risk allele. For rs4833095, T allele in *TLR1* conferred an increased risk in patients with IgAN in an allele model (OR = 1.27, 95% CI = 1.01–1.58, *P* = 0.04). However, there were no significant differences in the allelic distribution of the rs5743557 between the cases and controls (*P* = 0.13, Table 2). Genotype frequencies of rs5743557 genotypes CC, CT, and TT in the control group were 31.0%, 48.7% and 20.3%, respectively, and in the case group were 26.2%, 49.9% and 23.9%, respectively. Significant differences were not observed between the IgAN cases and healthy controls. The genotype distributions were not correlated with IgAN risk with statistic differences, though the OR values showed an increased risk trend of developing IgAN. For rs4833095, the genotype frequencies of CC, CT, and TT in the control group were 42.6%, 45.5%, and 11.9%, respectively, and in the case group were 35.3%, 49.0%, and 15.7%, respectively. The risk of developing IgAN was increased 40% after adjustment by age and gender in the dominant model (OR = 1.40; 95% CI = 1.02–2.13; *P* = 0.04), and was also significantly correlated with IgAN both before and after adjustment by age and gender in log-additive model (*P* = 0.038 and 0.03, respectively, Table 2).

**Table 2:** The associations between *TLR1* SNPs and IgAN

Model	Genotype	Control	Case	Before adjusted		After adjusted		AIC	BIC
				OR (95% CI)	*P*-value	OR (95% CI)	*P*-value		
**rs4833095 Codominant**	**C/C**	132(42.6%)	124(35.3%)	1.00	0.12	1.00	0.10	589.7	612.1
	**C/T**	141(45.5%)	172 (49%)	1.30(0.93-1.81)		1.36(0.98-2.11)			
	**T/T**	37 (11.9%)	55 (15.7%)	1.58(0.98-2.57)		1.53(0.91-2.90)			
**Dominant**	**C/C**	132(42.6%)	124(35.3%)	1.00	0.056	1.00	0.04	587.8	605.8
	**C/T-T/T**	178(57.4%)	227(64.7%)	1.36(0.99-1.86)		1.40(1.02-2.13)			
**Recessive**	**C/C-C/T**	273(88.1%)	296(84.3%)	1.00	0.16	1.00	0.26	589.6	607.6
	**T/T**	37 (11.9%)	55 (15.7%)	1.37(0.88-2.15)		1.29(0.82-2.34)			
**Overdominant**	**C/C-T/T**	169(54.5%)	179 (51%)	1.00	0.37	1.00	0.23	589.4	607.3
	**C/T**	141(45.5%)	172 (49%)	1.15(0.85-1.56)		1.22(0.91-1.83)			
**Log-additive**	---	---	---	1.27(1.01-1.59)	0.038	1.25(1.04-1.65)	0.03	587.9	605.8
**Allele**	**C**	405	420	1.27(1.01-1.58)	0.04	---	---	---	---
	**T**	215	282			---	---	---	---
**rs5743557 Codominant**	**C/C**	96(31%)	92(26.2%)	1.00	0.31	1.00	0.39	590.4	612.9
	**C/T**	151(48.7%)	175(49.9%)	1.21(0.84-1.73)		1.18(0.73-1.89)			
	**T/T**	63 (20.3%)	84 (23.9%)	1.39(0.90-2.15)		1.49(0.84-2.66)			
**Dominant**	**C/C**	96 (31%)	92 (26.2%)	1.00	0.18	1.00	0.30	589.3	607.2
	**C/T-T/T**	214 (69%)	259(73.8%)	1.26(0.90-1.77)		1.26(0.81-1.97)			
**Recessive**	**C/C-C/T**	247(79.7%)	267(76.1%)	1.00	0.26	1.00	0.23	588.9	606.9
	**T/T**	63 (20.3%)	84 (23.9%)	1.23(0.85-1.79)		1.35(0.82-2.21)			
**Overdominant**	**C/C-T/T**	159(51.3%)	176(50.1%)	1.00	0.77	1.00	0.96	590.3	608.3
	**C/T**	151(48.7%)	175(49.9%)	1.05(0.77-1.42)		0.99(0.66-1.48)			
**Log-additive**	---	---	---	1.18(0.95-1.47)	0.13	1.22(0.91-1.62)	0.18	588.5	606.5
**Allele**	**C**	343	359	1.18(0.95-1.47)	0.13	---	---	---	---
	**T**	277	343			---	---	---	---

Percentage of typed samples: 661/661 (100%); OR: odds ratio; 95% CI: 95% confidence interval; AIC: Akaike information criterion; BIC: Bayesian Information Criterion; Adjusted by age and gender.

### Haplotype analysis between patients with IgAN and healthy controls

We performed haplotype analysis for the two *TLR1* SNPs and susceptibility to IgAN. The haplotype frequencies are listed in Table 3. We found that the CC haplotype was more frequent in patients with IgAN. Further analysis of each of these haplotypes and the susceptibility to IgAN revealed that the TT haplotype (the minor alleles of rs4833095 and rs5743557, respectively) may be a risk factor for IgAN (OR = 1.28; 95% CI = 1.01–1.63; *P* = 0.046). The other haplotypes did not correlate with IgAN risk (*P* = 0.86 for CT haplotype and 0.84 for TC haplotype, respectively, Table 3).

**Table 3:** Haplotype analysis of *TLR1* and the associations with the risk of IgAN

rs4833095	rs5743557	Frequency	OR (95% CI)	*P*-value
C	C	0.502	1.00	---
T	T	0.347	1.28 (1.00 - 1.63)	0.046
C	T	0.122	0.97 (0.68 - 1.37)	0.86
T	C	0.029	1.07 (0.55 - 2.09)	0.84

OR: odds ratio, 95% CI: 95% confidence interval.

### Associations between genotype distribution and IgAN clinical variables

There were no statistical correlations identified between genotype distributions of rs5743557 and clinical parameters such as gender, 24 hour urine protein, blood pressure, and Lee's grades. However, rs4833095 was associated with Lee's grades (OR = 1.75; 95% CI = 1.03–2.96; *P* = 0.04), but not with gender, 24 hour urine protein, and blood pressure (Table 4).

**Table 4:** Genotype association analysis of *TLR1* variants with clinical parameters of IgAN

Variables	rs4833095				rs5743557			
	C/C (%)	C/T-T/T (%)	*P*	OR (95%CI)	C/C (%)	C/T-T/T (%)	*P*	OR (95%CI)
**Gender**								
** Female**	41 (33.6%)	81 (66.4%)		1.00 (reference)	33 (27.1%)	89 (72.9%)		1.00 (reference)
** Male**	83 (36.2%)	146 (63.8%)	0.62	0.89 (0.56-1.41)	59 (25.8%)	170 (74.2%)	0.79	1.07 (0.65-1.76)
**Urine protein (g/24h)**								
** <3.5**	101 (37.1%)	171 (62.9%)		1.00 (reference)	76 (27.9%)	196 (72.1%)		1.00 (reference)
** ≥3.5**	23 (29.1%)	56 (70.9%)	0.19	1.44 (0.84-2.45)	16 (20.2%)	63 (79.8%)	0.17	1.53 (0.83-2.81)
**Blood Pressure (mmHg)**								
** <140/90**	67 (34.5%)	127 (65.5%)		1.00 (reference)	47 (24.2%)	147 (75.8%)		1.00 (reference)
** ≥140/90**	57 (36.3%)	100 (63.7%)	0.73	0.93 (0.60-1.44)	45 (28.7%)	112 (71.3%)	0.35	0.80 (0.49-1.28)
**Lee's grades**								
** I+II+III**	100 (38.5%)	160 (61.5%)		1.00 (reference)	66 (25.4%)	194 (74.6%)		1.00 (reference)
** IV+V**	24 (26.4%)	67 (73.6%)	0.04	1.75 (1.03-2.96)	26 (28.6%)	65 (71.4%)	0.06	1.80 (0.98-3.30)

OR: odds ratio, 95% CI: 95% confidence interval.

*P* value was calculated from two-sided chi-square test.

## DISCUSSION

TLRs are a family of receptors that belong to type I membrane glycoproteins. TLRs can induce the differentiation of immune cells to regulate the immune system by generating different cytokines and can eventually cause tissue damage and a cascade of inflammations. TLRs are highly conserved from *Drosophila* to humans, sharing structural and functional similarities. Among TLRs, *TLR1* is ubiquitously expressed and is expressed at higher levels than other TLR family members. In humans, *TLR1* is located on chromosome 4p14 and encodes seven exons.

In the kidney, *TLR1* is expressed in glomerular mesangial cells [24] and tubular epithelial cells to activate the immune responses during tubulointerstitial injuries [25]. Several studies have assessed the association of *TLR1* gene polymorphisms with diseases. Hawn et al. reveals that *TLR1* G1805T variant is correlated with urinary tract infection in adult women [26]. Wurfel et al. reported that a *TLR1* variant is associated with increasing risk of organ dysfunction, death, and gram-positive infections in sepsis [27]. Recently, Mark et al. showed that the *TLR1* minor allele frequencies, p.His305Leu and p.Asn248Ser, are correlated with the prevalence of end-stage renal disease [28]. Hamann et al. found that rs4833095 involved in predisposition and manifestation of malaria during pregnancy [29]. Kim et al. indicated that there was no link between rs5743557 polymorphism of *TLR1* with benign prostatic hyperplasia in Korean descents [30]. To our knowledge, there are no studies assessing the relationship between *TLR1* variants and IgAN in Chinese Han populations, and only one study showing a link between *TLR1* gene polymorphisms and childhood IgAN [23]. Therefore, we performed this case-control study to investigate the relationship between two *TLR1* SNPs (rs4833095 and rs5743557) and IgAN in Chinese Han patients. Our results indicate that the allele frequencies of rs4833095 were associated with increased IgAN risk. The allele and genotype frequencies of rs5743557 were both not correlated with IgAN susceptibility in six genetic models. However, the genotype frequencies of rs4833095, in the log-additive model, were associated with increased risk of IgAN.

In contrast, Lee et al. demonstrated that rs4833095 and rs5743557 were associated with childhood IgAN susceptibility in Korean population [23]. Here, the allele frequencies of T and C in rs4833095 were 34.7% and 65.3% in the control group and 40.2% and 59.8% in IgAN group. Our results are similar to those observed in Korean populations, where the frequencies of T and C alleles in the control group were 42.4% and 57.6%, respectively and in the IgAN group were 37.9% and 62.1%, respectively. In our study, the major allele of rs4833095 was the C allele, which was also the case in African American, Sub-Saharan African, and Asian populations, but not in European populations. While the study by Lee et al. identified that rs4833095 C allele frequency was significantly increased in IgAN in Korean patients. Our results differed from them that C allele frequency was a little decreased in Chinese IgAN patients. Besides, our results revealed that the genotype frequencies of rs4833095 were associated with increased risk of IgAN in the dominant model and log-additive model. This was partly in conjunction with the previous result [23]. Furthermore, the *TLR1* rs4833095 polymorphism was found to affect the signaling functions of *TLR1* in patients with infectious disease [31]. The T allele was observed to weaken the immune response of *TLR1* to agonists, and the C allele might strengthen the normal functions of *TLR1* [31–32] Therefore, rs4833095 gene variant C substitution to T in patients with IgAN might contribute to the changes in *TLR1* signaling functions, which may weaken the immune reactions to pathogens and other agonists. Eventually, this phenomenon may lead to cellular and pathological injuries in patients with IgAN, such as mesangial cell proliferation, accumulation of extracellular matrix and excessive deposition of IgA1. We speculate that immune imbalances in patients with IgAN may accelerate the progression to end stage renal disease and have poor outcomes and prognosises. Furthermore, we thought the functional consequences of rs4833095 of *TLR1* gene in patients with IgAN may ascribe to the up-regulated transcription and protein expressions of *TLR1* and its underlying signaling pathways, such as MAPK and NF-κB.

In our study, the allele frequencies of rs5743557 C and T were consistent with those observed in Korea [23]. The major allele detected in our study was the C allele, which was similar with the Korean population. Additionally, the allele and genotype distributions of rs5743557 in this study were not associated with IgAN risk. The Korea study identified that rs5743557 C allele frequencies were increased in IgAN groups, though after Bonferroni correction this association was no longer statistically significant. The Korean study also found that the TT genotype of rs5743557 to be associated with increased risk of childhood IgAN compared with the CC genotype in the codominant model.

Our haplotype analysis indicated that the TT haplotype might be a risk factor for IgAN. This result is different from that of the Korean study, in which the CT haplotype was correlated with IgAN. Different environment, background, ethnicity, population mixture, and age groups may account for these inconsistencies.

We assessed the relationship between *TLR1* rs4833095 and rs5743557 genotype distributions and gender, 24 hour urine protein, blood pressure, and Lee's grade and IgAN. Consistent with the Korean study, no obvious statistical differences were observed for the rs5743557 SNP and these parameters in our study [16]. However, rs4833095 was associated with Lee's grade. Discrepancies between our results and those of the Korean study may be ascribed to differences in patient parameters such as age, clinical manifestation, therapeutic schemes, and different standards for responder and non-responder subjects.

To our knowledge, this is the first report regarding the relationships between rs4833095 and rs5743557 polymorphisms and IgAN and its clinical variables in *TLR1* in a Chinese Han population. And the number of IgAN patients in our study was 351, which was larger than the Korean 190 IgAN patients. Furthermore, subgroup analysis indicated that the rs4833095 polymorphism was significantly associated with Lee's grades, which may be indicative of the disease severity. However, functional studies of *TLR1* in patients with IgAN are still needed in the future to evaluate the precise relationship between *TLR1* SNPs and the developing or progression and prognosis of IgAN.

Limitations of our study included an incomplete follow-up duration and sample size. The sample size of our study was small, and it may not have had enough statistical power to determine real correlations. Last but not least, we lack functional studies in our present study. To reach a more accurate conclusion, additional gene-environment and gene-ethnicity interaction studies with larger sample sizes are required.

This study demonstrated that the *TLR1* variant rs4833095 was correlated with the pathological conditions and the risk of IgAN. Additionally, the T _rs4833095_T _rs5743557_ haplotype of both and was correlated with increased risk of IgAN. Taken together, these results indicate that *TLR1* gene polymorphisms may play a role in the development and / or progression of IgAN in the Chinese Han population.

## MATERIALS AND METHODS

### Ethics statement

The study protocol was approved by the ethics committee of the Second Affiliated Hospital of Xi'an Jiaotong University. Written informed consent was obtained from all participants after a full explanation of the study. The experimental protocol was implemented in accordance with the approved guidelines.

### Subjects

This hospital-based case-control study was assessed by using the Newcastle-Ottawa scale [33] and performed by enrolling 351 patients with IgAN (229 males and 122 females, mean age of 32 ± 11.9 years) from Northwestern China at the First and Second Affiliated Hospital of Xi'an Jiaotong University from March 2009 to April 2014. The diagnosis of IgAN was confirmed by renal biopsy. The controls were 310 healthy subjects (186 males and 124 females, mean age of 35 ± 12.6 years) recruited from routine healthy examinations in the same hospitals. All subjects were from the Chinese Han population living in Xi'an city or nearby. Patients with comorbidities such as diabetes mellitus, lupus nephritis, and other secondary IgAN were excluded. Demographic and clinical details were collected, including age, gender, 24 hour urine protein, blood pressure, serum creatinine level (SCr), blood urea nitrogen (BUN), serum albumin level (ALB), serum cholesterol level (Cho), serum IgA level, serum C3 level, and histopathological grade (Lee's classification). The definition of Lee's grades and the renal histological lesions were evaluated using this criteria [34] and were listed below. I: Most glomerulars are normal and without tubular and interstitial changes. II: Slightly thicken mesangium with segmental distribution in glomerular and absent of tubular and interstitial changes. III: Diffuse mesangial cell proliferations and thicken mesangium with focal and segmental distribution in glomerular, sometimes could see crescents and adhesions. Focal interstitial edema and imflammation cells infiltrate occasionally shown in tubular and interstitial areas with or without tubular atrophy. IV: Sharply diffuse mesangial cell proliferations plus partial or total sclerosis, and up to 45% glomeruli with crescents in glomerular. Tubular atrophy, inflammation and occasionally appears foam cells in tubular and interstitial. V: More than 45% glomeruli appear crescents and the lesions in glomerular, tubular and interstitial areas are more severe than IV.

### DNA extraction and genotyping

Blood samples were collected in tubes containing ethylene diaminetetraacetic acid (EDTA). After centrifugation at 1,500 rpm for 10 min, the samples were stored at −80°C. Genomic DNA from whole blood was extracted using the GoldMag DNA Purification Kit (GoldMag Co. Ltd, Xi'an City, China), and the purity and concentration was measured utilizing an ultraviolet spectrophotometer (Nanodrop, Thermo Scientific, Waltham, MA). We searched *TLR1* gene SNPs in the NCBI dbSNP. Some of the SNPs were excluded due to the MAF were < 0.1 or the absence of genotype distribution data in Asian population. Eventually, combined with the current studies, two common polymorphisms (rs4833095 and rs5743557) in *TLR1* gene were selected in the present study. We also calculated the statistical power of rs4833095, which was 0.86. This showed that our sample size was adequate and the results were acceptable. The Sequenom MassARRAY Assay Design 3.0 software was used to design Multiplexed SNP MassEXTEND assay. SNP genotyping was performed by using Sequenom MassARRAY RS1000 according to the standard protocol. The primers used for rs4833095 and rs5743557 are listed in Table 5. Sequenom Typer 3.0 software was used for data analysis.

**Table 5:** Primers used for this study

SNP_ID	1st-PCRP	2nd-PCRP	UEP_SEQ
**rs4833095**	ACGTTGGATGCCTAAG TATTCTGGCGAAAC	ACGTTGGATGCTGGAG GATCCTAATGAAAG	TTCAATGTTGTTTAAGGTAAGA
**rs5743557**	ACGTTGGATGGTGTGT TTCGGCTGCTTTGT	ACGTTGGATGACTTTAA TCTCGACCCCTCC	GACCCCTCCCTCTTT

### Statistical analysis

Data analysis was performed using SPSS 18.0 statistical package (SPSS, Chicago, IL, USA). The SNP frequency in the controls was assessed for departure from Hardy–Weinberg Equilibrium (HWE) using an exact test. We calculated allele and genotype frequencies of cases and controls using a χ^2^ test. Odds ratios (ORs) and 95% confidence intervals (CIs) were determined using unconditional logistic regression with adjustment for age and gender. Six genetic models (codominant, dominant, recessive, overdominant, log-additive and allele) were used to evaluate potential association of *TLR1* polymorphisms with risk of IgAN. Statistical power was calculated was by Power and Sample Size Calculation software. Haploview software was used to conduct haplotype analysis. *P* < 0.05 was considered statistically significant and all statistical tests were two-sided.
